# Differences in clinical features and risk factors for striae distensae in Black and White women

**DOI:** 10.1007/s00403-025-04050-z

**Published:** 2025-03-18

**Authors:** Nada Elbuluk, Autumn L. Saizan, Arielle Carolina Mora Hurtado, Ted Hamilton, Sewon Kang

**Affiliations:** 1 Department of Dermatology, Keck School of Medicine of University of Southern California, Los Angeles, CA USA; 2 Department of Dermatology, Johns Hopkins University School of Medicine, Baltimore, MD USA; 3 University of Wisconsin School of Medicine and Public Health, Madison, WI USA; 4 Department of Dermatology, University of Michigan Medical School, Ann Arbor, MI USA

**Keywords:** Ethnicity, Race, Skin of color, Striae alba, Striae distensae, Striae rubra

## Abstract

Striae distensae (SD) are a common condition, which can appear differently across skin colors and for which effective treatments remain limited. SD have several risk factors, including pregnancy, obesity, growth spurts, and several pathologic conditions. Few studies have examined whether there are skin color differences regarding SD in their clinical presentation, risk factors, and associated comorbidities. To evaluate the clinical features, risk factors, and associated comorbidities of SD among Black and White women. This was a two-part study involving a telephone questionnaire followed by an in-person clinical assessment with standardized photographs. One hundred forty-three women (75 Black, 68 White) completed the survey, and 66 women (33 Black, 33 White) completed the in-person clinical assessment. Black and White women in the study were found to be similar in age, SD duration, parity, pregnancy-associated weight gain, and family history. Black women, on average, had a greater number of SD than White women (118 versus 76, *p* = 0.01). Striae were typically white and skin-colored among Black women, but white and violaceous among White women (*p* = 0.02). Black women were more likely to have involvement of the lower legs (*p* = 0.04), axilla (*p* = 0.05), and buttocks (*p* = 0.002) than White women. Compared to Black women, urinary incontinence was more commonly reported among White women, though this did not reach statistical significance (*p* = 0.07). There was a significant association between smoking and SD in White women (*p* = 0.003), but not in Black women. Additionally, Black women were more likely to use creams to diminish the appearance of their striae. While the etiology, prevalence, and risk factors of SD may be similar between Black and White women, there may be important skin color differences in SD clinical features and medical comorbidities. Larger studies are needed to further characterize the relationship between SD and medical comorbidities such as urinary incontinence and pelvic floor dysfunction. The study of this relationship may advance understanding of SD pathogenesis and provide pathways for targeted therapies. More studies are needed to determine the role of SD evaluation as a screening tool to help predict the risk of the development of pelvic floor dysfunction.

## Introduction

Striae distensae (SD) are a common dermatologic condition that may initially appear as multiple, linear erythematous to violaceous patches or plaques and may later transition to atrophic, hypopigmented scars with fine wrinkling [1]. While the exact pathogenesis is not fully elucidated, it has been postulated that SD is associated with alterations in the components of the extracellular matrix, such as fibrillin, elastin, and collagens, which provide tensile strength to the skin [2]. Genetic factors and hormonal influences are also thought to contribute to the pathogenesis of SD [3]. SD have a multitude of risk factors, including genetics, family history of striae, neonatal weight, mechanical skin tension, medications, and hormones [1, 2, 4–6]. Physiologic changes such as puberty growth spurts, pregnancy, rapid fluctuations in weight, or pathologic states, such as Cushing’s disease/syndrome, liver disease, and genetic conditions may all contribute to striae formation [1, 5–7]. Medications, including topical and systemic corticosteroids, also serve as potential etiologies [1, 5, 6].

Appearance may vary by skin color as well as underlying etiology or physiologic state. Duration and stage of the SD can play a factor in their appearance. Previous reports in the literature note that individuals with darker skin types typically present with brown to black striae, while those with lighter skin types present with red to purple striae that gradually become white over time [8]. Amongst those with hypercortisolism or Cushing’s disease, striae may appear blue [8].

SD are most prevalent among women, particularly those that are pregnant [1]. They are also common among adolescents and young adults [8, 9]. The ocation of SD often depends on patient sex and associated physiologic state. After puberty, the breasts, buttocks, thighs, and calves are more commonly affected in females, while the lower back, outer thighs, and knees are more often affected in males [1, 9]. In pregnant women, the breast, thighs, and abdomen are usually involved [1]. Although typically asymptomatic, some patients report associated pruritus [10]. Patients may also present with significant psychological distress and cosmetic concern given the potentially disfiguring nature of the condition [10, 11]. There is currently no adequate therapeutic approach for treating striae. Topical retinoids, glycolic acid, L-ascorbic acid, succinylated collagen, herbal extracts (*centella asiatica*), hyaluronic acid, silicone gel, chemical peels, carboxytherapy, platelet-rich plasma therapy, microdermabrasion, subcision, radiofrequency, radiofrequency microneedling, and various light lasers have shown varying success. Studies remain limited, particularly across patients with diverse skin colors [2, 6, 12, 13]. The treatment of striae in patients with skin of color is further complicated by risk of post-inflammatory hyperpigmentation secondary to procedural therapies [13].

Few studies have documented variation in striae appearance by demographics, risk factors, and associated comorbidities. The primary aim of this study was to examine differences in the prevalence and clinical characterization of striae as well as the associated risk factors and comorbidities among White and Black women.

## Methods

This was a single-center, two-part prospective study consisting of a brief telephone interview and subsequent, optional in-person clinical evaluation. The study was approved by the University of Michigan Medical Institutional Review Board. All participants provided informed consent. Patients were evaluated based on demographics, clinical features, and potential risk factors. Telephone surveys and clinical exams were conducted by clinicians and investigators from the University of Michigan’s Department of Dermatology.

This study involved women ages 35–70 located in Southeast Michigan. All patients were recruited from a database previously created by the Department of Obstetrics and Gynecology. This database included 394 patients who participated in a prior study on racial differences in female urinary incontinence. Eligible individuals for this striae study were female, 18 years of age or older, in good general health, and able to follow the protocol and provide written informed consent. The exclusion criteria included patients receiving an experimental drug or using an experimental device 14 days prior to study enrollment, as well as patients with disease states or physical conditions impairing evaluation at the test sites. A letter discussing the purpose of the study, what the study would involve, and the ability to opt out was sent to all eligible participants by mail. Those not opting out of the study were contacted by phone for survey completion. No financial incentives were offered for participation.

For the first part of this study, a ten-minute survey was conducted by phone to obtain patient demographics, including age, race/ethnicity, height, and weight. The phone survey also queried patients about their age of menarche, presence of striae, location, duration of striae, cause of striae, parity, gestational weight gain, associated symptoms, and exercise frequency and type. Patient history of dermatologic conditions, chronic diseases, medications, surgical history, and family history of striae were also queried to determine potential contributing factors of SD.

After survey completion, patients were invited to participate in the second part of the study, which involved attending the dermatology clinic at the University of Michigan to have their striae clinically evaluated and photographed. During the clinical evaluation, number, location, morphology, and type of striae were assessed. A chromometer was used to assess skin pigmentation. Standardized photographs of affected areas were taken of each patient by a medical photographer.

### Statistical analyses

Differences in factors associated with striae in Black and White women were statistically compared using the two-sample t-test for continuous data and the chi-square test for categorical data. Correlations among variables were assessed with Pearson’s product-moment correlation coefficient or regression analysis methods. Statistical significance was attained with *P* ≤ 0.05 for a two-tailed hypothesis. Descriptive statistics are represented as mean ± SEM. The data were analyzed by SAS statistical software (v.9.1; SAS Institute, Inc., Cary, NC).

## Results

**Fig. 1:**
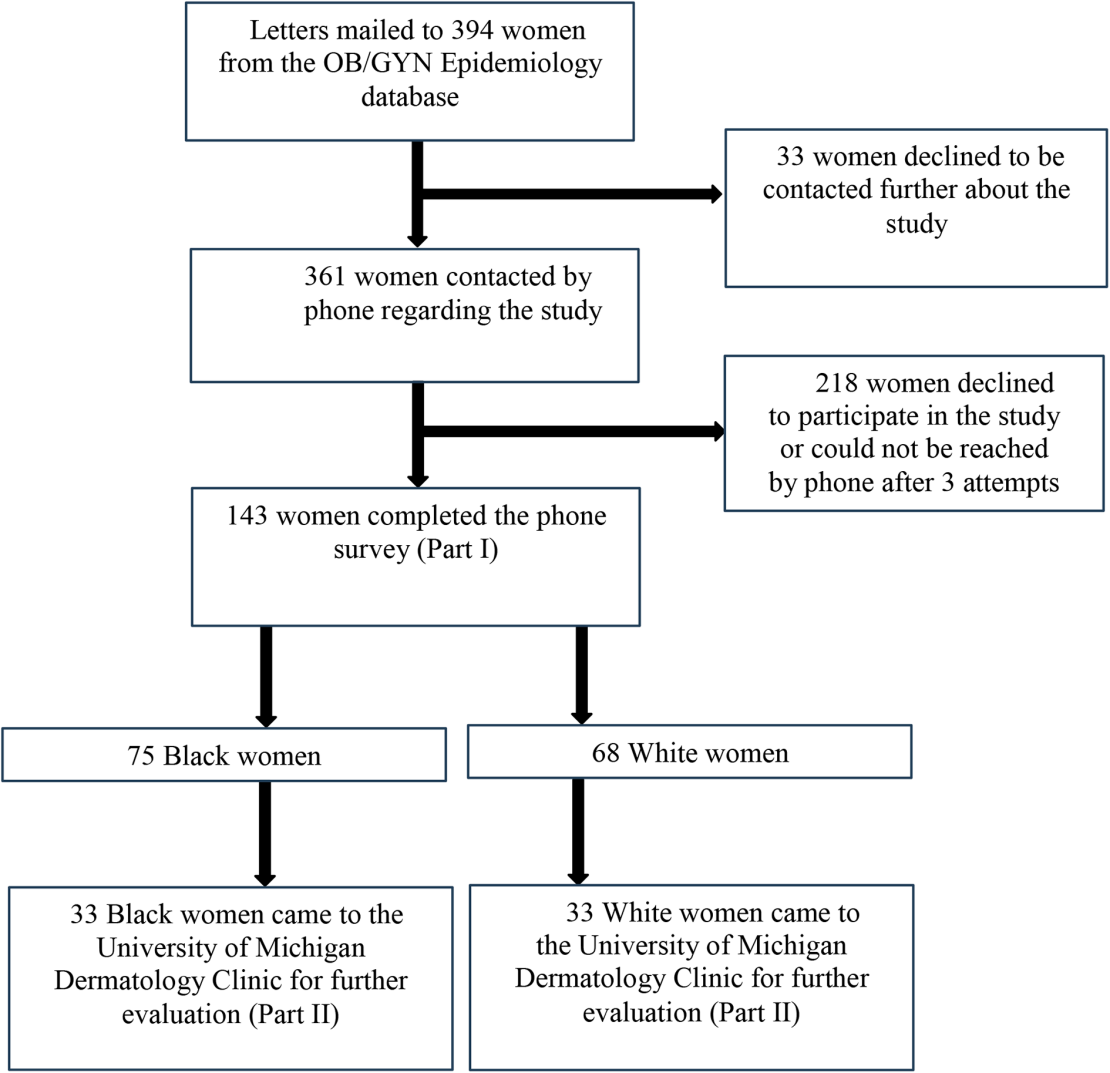
Flowchart of study recruitment method

Of the 394 eligible patients, a total of 143 patients completed the survey, resulting in a survey response rate of 36% (143/394). Figure 1 demonstrates the method for study recruitment and enrollment. Of the 143 patients who completed the survey portion of the study, 52.4% (*n* = 75) were Black and 47.6% (*n* = 68) were White. Of the 66 patients completing the in-person evaluation, half were Black (*n* = 33) and half were White (*n* = 33).

Across all patients (*n* = 143), the mean age was 55.5 ± 0.6 years and the mean BMI was 31.1 ± 0.7. Black and White women were similar in mean age, age at menarche, BMI, history of pregnancy, number of pregnancies, average gestational weight gain, and length of time with SD (Table 1).

**Table 1:** Striae survey responses among Black and White women

Subject Characteristics	Black Women	White Women	*P* value
	n = 75	n = 68	
Age (mean years)	56.0 ± 0.9	54.9 ± 0.9	NS, 0.70
Age at menarche (mean years)	13.1	12.7	NS, 0.069
Body mass index	32.2 ± 0.9	29.8 ± 0.9	NS, 0.066
History of pregnancy	56	46	NS, 0.70
Number of pregnancies (mean number)	2.48 ± 0.20	2.54 ± 0.28	NS, 0.849
Weight gained with pregnancy (mean lbs.)	30.1 ± 1.2	33.5 ± 2.9	NS, 0.312
Length of time with striae (mean years)	28 ± 1.6	25 ± 1.9	NS, 0.17
**Striae caused by**:			
Pregnancy	40	30	NS, 0.325
Non-pregnancy weight gain	15	11	NS, 0.622
Pregnancy and weight gain	47	43	NS, 0.622
Striae symptoms	29	20	NS, 0.290
Average number of striae	118 ± 13	76 ± 10	**0.013**
Number of body parts with striae	1.57	1.33	NS, 0.14
Number of body parts with striae in women with history of pregnancy	1.64	1.43	NS, 0.22
Striae on 4 or more body parts	17	5	**0.011**
White and violaceous striae	1	10	**0.015**
White and skin-colored striae	3	1	**0.015**
**Striae Locations**:			
• Lower legs	7	1	**0.041**
• Buttocks	28	10	**0.002**
• Axilla	4	0	**0.053**
• Stomach	54	44	NS, 0.348
• Arms	9	4	NS, 0.204
• Thighs	34	26	NS, 0.390
Striae creams used	16	7	**0.020**
**Past Medical History**			
Positive medical history	61	54	NS, 0.65
Positive family history of striae	55	43	NS, 0.251
Past surgical history	68	59	NS, 0.46
Past dermatologic history	19	23	NS, 0.27
Average weekly physical activity (mean hours)	1.71	2.48	NS, 0.08
**Comorbidities**			
Striae and urinary incontinence	33	36	NS, 0.333^1^ NS, 0.074
Striae and cardiac history	44	29	NS, 0.056
Striae and psychiatric history	7	20	**0.002**

NS: Not significant. Means are expressed as $\bar{\rm{x}}$ ± 1 SEM.

^1^The top p-value compares the association between incontinence and striae in Black women, the bottom value compares the association between incontinence and striae in Whites

There was no significant difference in the patient-reported causes of SD between Black and White women. These causes included pregnancy, non-pregnancy related weight gain, or a combination of both. No differences in the number of body parts with SD in those with a history of pregnancy were observed (*p* = 0.22). There were also no significant differences in medical history (*p* = 0.65), family history of striae (*p* = 0.24), surgical history (*p* = 0.46), and dermatologic history (eczema, rosacea, acne, keloids) (*p* = 0.27) between Black and White women. In regard to lifestyle factors, no difference in average weekly physical activity among Black and White women was seen (*p* = 0.08). Moreover, there were no significant differences in the presence of symptoms from striae (itching, burning, swelling, dryness, other) in Black and White women (*p* = 0.29).

White women had more psychiatric conditions than Black women (*p* = 0.002). Black women had a higher prevalence of cardiac conditions; however, the latter association did not reach statistical significance (*p* = 0.0556). Also, this study showed a near significant association between urinary incontinence and striae in White women (*p* = 0.07), which was not observed in Black women. When comparing White women smokers and non-smokers, there was a significant association between smoking and SD (*p* = 0.003). However, this association was not significant in Black women (*p* = 0.522). There was a moderate strength correlation between body mass index (BMI) and striae number (*r* = 0.45, *p* = 0.0002) as well as BMI and number of body parts with striae (*r* = 0.26, *p* = 0.0018).

**Fig. 2:**
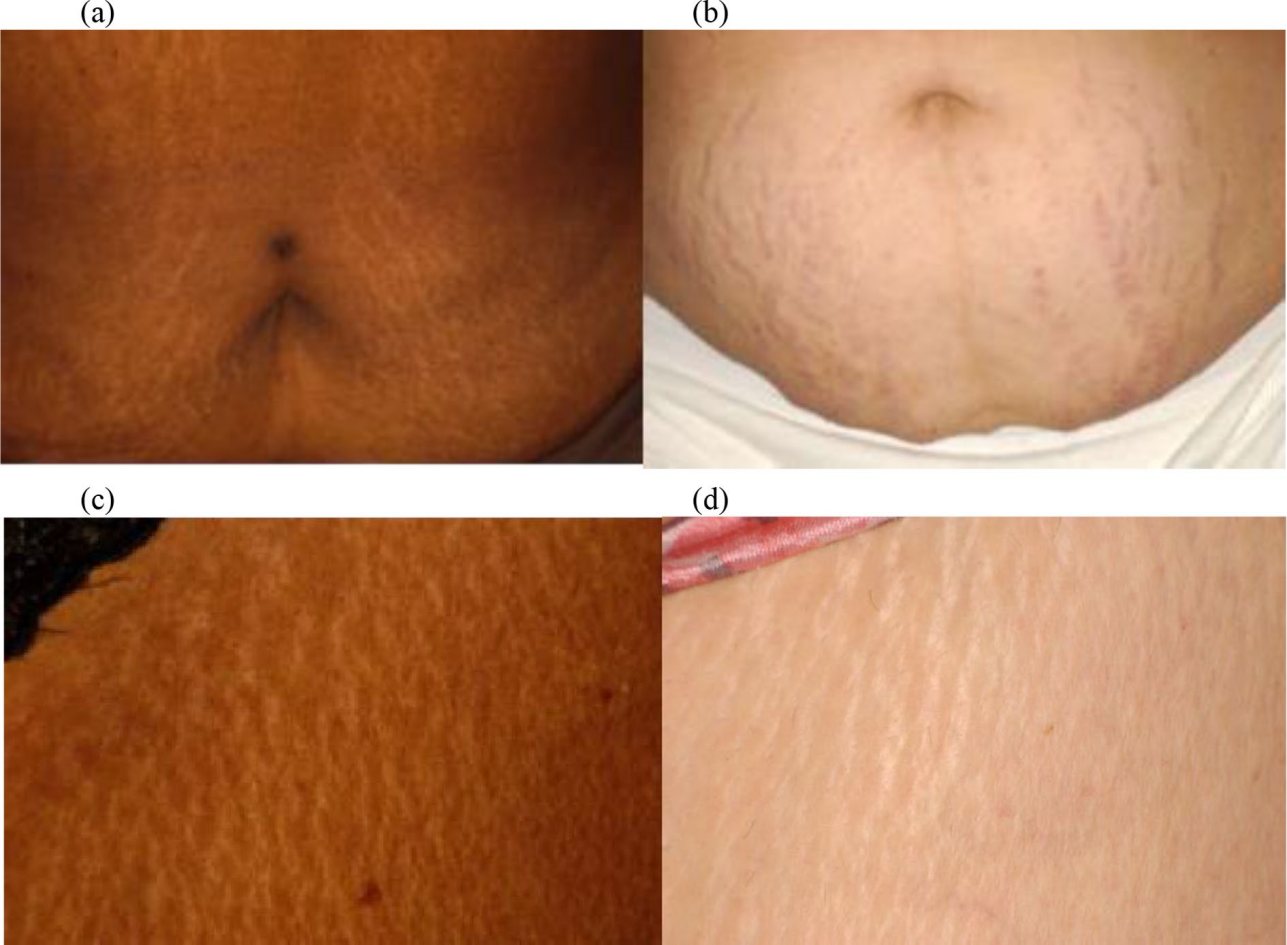
The top row compares white (hypopigmented) striae on the abdomen of a Black woman (**a**) with violaceous purple striae on the abdomen of a White woman (**b**). The bottom row shows white striae on the thigh of a Black woman (**c**) and on the thigh of a White woman (**d**)

**Fig. 3:**
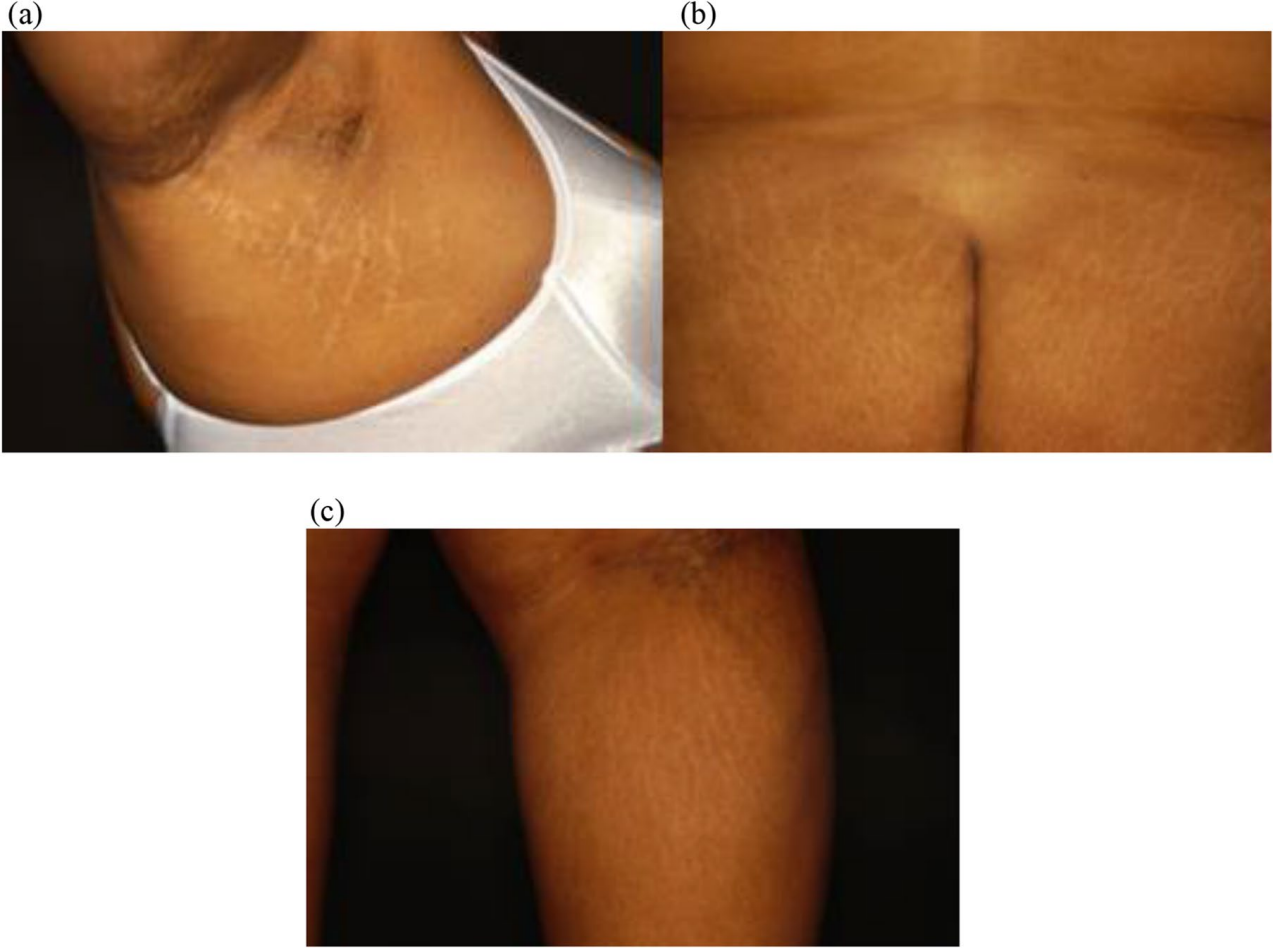
Black women had more striae in their axilla (**a**), buttock (**b**), and lower legs (**c**) than White women

**Fig. 4:**
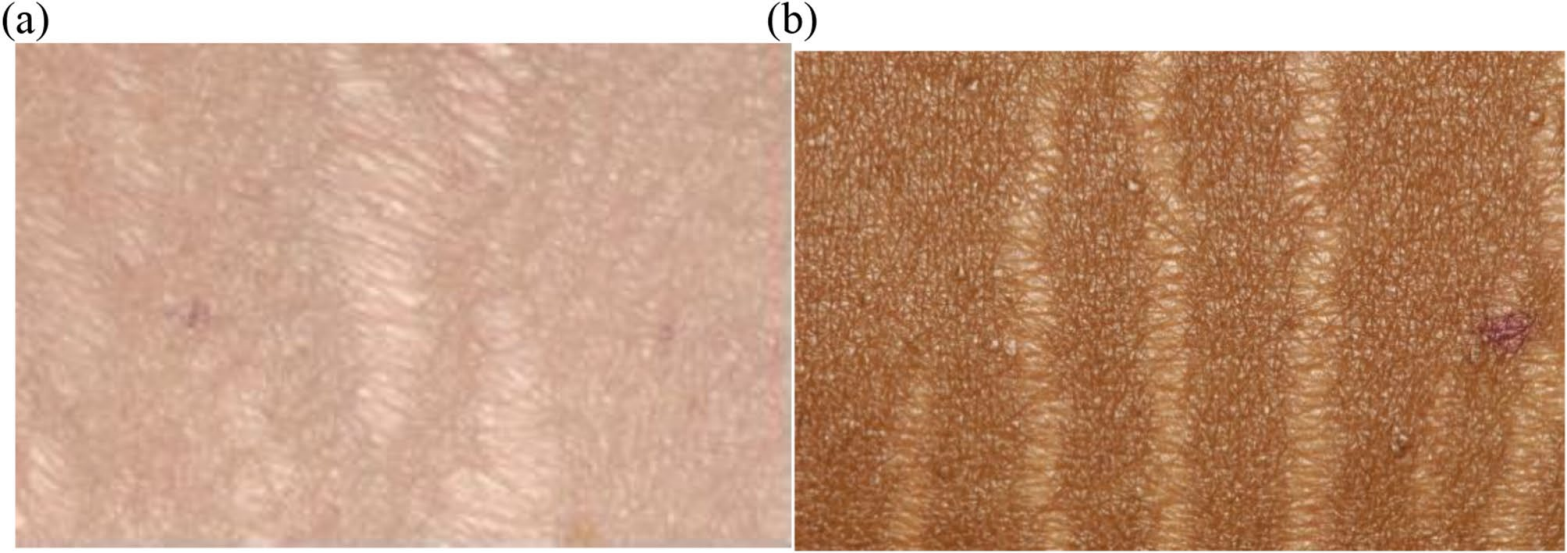
The images show the close-up appearance of hypopigmented, atrophic striae in a White woman (**a**) and a Black woman (**b**)

Black women on average had a significantly higher number of striae than White women (118 versus 76, *p* = 0.01) and were more likely to have striae on four or more parts of their body (*p* = 0.01). Striae tended to appear white and skin-colored in Black women, but white and violaceous in White women (*p* = 0.02) (Fig. 2). Compared to White women, Black women more often had striae on their lower legs (*p* = 0.04), axilla (*p* = 0.05), and buttocks (*p* = 0.002) (Figs. 3 and 4). No significant difference was found regarding the presence of striae on the stomach, arms, and thighs between these two groups.

Black women more frequently reported using topical creams to remove or diminish the appearance of their striae compared to White women (*p* = 0.02).

## Discussion

While the causes of striae are understood, few studies have formally evaluated the clinical features, risk factors, and associated comorbidities of SD in the context of skin color. This study highlights the variation in striae number, color, and location between Black and White women. This study also differentiates potential risk factors, such as high BMI, and associated comorbidities, such as urinary incontinence and smoking, among the two racial groups. With respect to striae color, White women were more likely to have white and violaceous striae, while Black women were more likely to have white and skin-colored striae. Hermanns and Pierard reported black-appearing striae, referred to as striae nigrae, in those with darker skin types [14]. Other variations of striae include striae rubrae (red) and striae albae (white), which often correspond to new and old lesions, respectively, and striae caerulae, which are typically blue and associated with hypercortisolism [7, 14]. Hermanns and Pierard suggest that hormonal, physiological, and mechanical factors ultimately contribute to variations in striae color via direct or indirect influence of dermal cell growth, genetic expression, vasodilation, angiogenesis, and melanocyte activity [14]. The erythema in striae rubrae may occur secondary to vasodilation and angiogenesis [6, 7, 14]. Erythema noted in striae nigrae suggests striae nigrae may occur in conjunction with striae rubrae in those with darker skin [13]. Striae albae are likely to occur with the resolution of mechanobiological stimuli and subsequent cessation of vascular stimulation and melanogenesis [8, 14]. Striae color therefore likely depends on staging, underlying conditions, and baseline skin color. Regardless of the clinical presentation or underlying etiology, histopathology remains consistent between the types of SD and typically resembles a scar [6–8, 15].

With respect to striae number and location, this study found that Black women had a significantly higher number of striae and number of affected body locations. Black women were more likely to have striae on four or more body parts, particularly their lower legs, axilla, and buttocks. Black women had a higher BMI than White women, although this data did not reach statistical significance. The higher number of striae could be related to having a higher BMI as there was a moderate association found between BMI and striae number as well as body distribution. A study focusing on body composition noted significantly higher BMIs in women with striae compared to women without striae [16]. They also noted significantly higher BMIs and other body composition factors, such as fat percentage, muscle mass, fat-free mass, and basic metabolic rate, in women with abdominal striae. Each of these values were significantly lower in women with striae limited to the buttocks, suggesting there is a greater association between a high BMI and abdominal striae than striae in other locations [16]. This study, however, was ethnically homogenous with only White women being included and therefore lacked generalizability [16]. Additional studies investigating how striae location may vary by race and by BMI in the context of race are needed.

With respect to risk factors, our study did not find differences between family history, personal history, and/or weight gain and associated striae formation between the two groups of women. Age, duration of striae, number of pregnancies, and amount of gestational weight gain were similar between Black and White women. Our study suggests that causes or risk factors for striae are unlikely to differ between Black and White women. A review of the literature, however, reveals inconsistent results. Poidevin et al. found no association between the degree of abdominal stretch during pregnancy and the number of striae, suggesting genetics may play a greater role in striae formation than mechanical stretch [17]. This is further supported by another study that found that women with striae gravidarum (SG) had a higher prevalence of vaginal lacerations during childbirth than those without SG, proposing a genetic component in how the skin responds to mechanical stress [18]. Chang et al. proposed that family history of striae, personal history of striae, and race may serve as better predictors of SG development than pre-pregnancy or pregnancy weight gain [10]. Nevertheless, some studies report more striae or greater striae severity with mechanical stretch secondary to higher maternal weight, infant birth weight, and parity [19, 20]. Other studies note increased prevalence amongst those with high BMIs and short stature [15, 21]. Inconsistent results between studies support the notion that the risk factors for SD are multifactorial and often concurrent. Genetics, mechanical stretching, hormones, and body habitus may all influence striae formation, perhaps to varying degrees in the context of race and ethnicity.

Our study found no difference in striae prevalence between study participants and the general Black and White populations or Black and White women who have been pregnant. Chang et al. found a higher prevalence of SG in women of color, particularly among African American, Hispanic, East Asian, and South Asian individuals [10, 22]. This is contradicted, however, by Poidevin et al., who found a higher prevalence of striae in those with lighter skin, although participant race was not reported [10, 17]. In a study consisting of only Afro-Caribbean women, striae prevalence was roughly 50%, which is similar to the prevalence in other studies consisting of primarily White women [23–25]. Whether or not there is a difference in the prevalence of striae among certain racial/ethnic groups remains unclear. It is likely that race/ethnicity and skin color have less of an influence on striae risk factors or prevalence and more of an influence on striae color, number, and location.

Striae may be associated with several comorbidities. Previous studies report striae and greater striae severity, in terms of number and distribution, as significant risk factors for pelvic organ prolapse and its associated symptoms [20, 26, 27]. The association of striae and pelvic organ prolapse may represent a shared genetic factor resulting in connective tissue defects [27]. Previous studies note a common pathogenesis between striae and pelvic organ prolapse [20, 27]. In a study involving 108 women, Salter et al. demonstrated an association between striae and pelvic organ prolapse that was significant even after controlling for age, weight, number of pregnancies, preterm labor, menopausal status, or history of hemorrhoids, varicose veins, and systemic steroid use [26]. In this same study, multivariate logistic regression identified the presence of striae as a predictor for clinical pelvic prolapse (odds ratio 3.12) [26]. Further, in a cross-sectional study involving 110 women, after adjusting for confounders, women with pelvic organ prolapse were found to have 2.5 times higher odds of having striae compared to women without pelvic organ prolapse [28]. Findings from these studies suggest that striae may serve as a risk factor for the development of pelvic floor disorders [26–28].

In the present study, urinary incontinence, a common symptom of pelvic floor dysfunction, was more common among White women compared to Black women, though this did not reach significance. In a study by Graham and Mallet, findings suggested that White race may be a significant predictor of genuine stress urinary incontinence [29]. Our study and review of the literature suggest that race and the presence of striae may be predictive of pelvic floor dysfunction subtypes. Pure genuine stress incontinence is reportedly more prevalent among White women and pure motor incontinence/detrusor instability among Black women [29, 30]. The presence of striae may provide predictive value for the development of pelvic organ prolapse and may provide an opportunity for patient education and counseling on lifestyle modification to decrease the risk of adverse sequelae [26, 27]. This includes quitting smoking, which was significantly associated with striae development among White women in our study [27]. Other lifestyle modifications to reduce the risk of pelvic floor dysfunction include reducing coffee consumption, avoiding heavy lifting and straining, and reducing excess weight [26, 27]. Larger investigative studies should assess racial/ethnic differences across skin colors in the association between striae and pelvic organ prolapse development, accounting for confounding factors, such as the route and duration of vaginal delivery [27, 29]. The relationship between striae and other conditions, such as low back pain during pregnancy, obstetric anal sphincter injuries, preterm deliveries, perineal trauma, and intraabdominal adhesions, has also been assessed [31–36].

Finally, striae, along with the lack of effective treatment can negatively impact mental and emotional health, leading to decreased quality of life [6, 11]. In our study, Black women were more likely than White women to use creams to diminish the appearance of their striae. This may be because striae in Black women are more noticeable and/or cosmetically bothersome, and thus, there may be an increased desire to reduce the appearance of striae among this population.

### Limitations

Our study is limited by selection bias, as women were recruited from an initial sample of women participating in a study about female urinary incontinence attending a gynecology clinic. In addition, this was a single-center study involving predominantly middle-aged patients with a high BMI. The population of patients visiting the University of Michigan facilities may not be generalizable to other populations. Furthermore, the survey portion of the study was limited by patients’ ability to recall their medical history and the use of an unvalidated questionnaire.

## Conclusion

While the etiology, prevalence, and risk factors of striae appear to be similar between Black and White women, there may be important differences in striae appearance, number, distribution, and medical comorbidities in light and dark skin. This study showed an association between striae and smoking, as well as a nearly significant association between striae and urinary incontinence in White women compared to Black women. Larger studies examining the relationship between striae and medical comorbidities such as pelvic organ prolapse are warranted. Elucidating this relationship between SD and pelvic organ prolapse, two disorders involving altered collagen function, could advance the understanding of striae pathogenesis and offer new therapies for striae, which are currently limited. More specifically, these treatment modalities may include those that stimulate collagen production [37]. In addition, more study is needed to determine the role of striae evaluation as a screening tool, helping to predict patient risk for the development of certain conditions such as pelvic organ prolapse. This knowledge may facilitate patient counseling, influence treatment plans, and ultimately improve patient outcomes.

## Data Availability

The data that support the findings of this study are available from the corresponding author upon reasonable request.

## Abbreviations

SD: Striae distensae
SG: Striae gravidarum
